# Acquired bloodstream infection in the intensive care unit: incidence and attributable mortality

**DOI:** 10.1186/cc10114

**Published:** 2011-03-21

**Authors:** John R Prowle, Jorge E Echeverri, E Valentina Ligabo, Norelle Sherry, Gopal C Taori, Timothy M Crozier, Graeme K Hart, Tony M Korman, Barrie C Mayall, Paul DR Johnson, Rinaldo Bellomo

**Affiliations:** 1 Department of Intensive Care, Austin Hospital, 145 Studley Road, Heidelberg, Victoria 3084, Australia; 2 Department of Infectious Diseases, Austin Hospital, 145 Studley Road, Heidelberg, Victoria 3084, Australia; 3 Department of Intensive Care, Monash Medical Centre, 246 Clayton Road, Clayton, Victoria 3168. Australia; 4 Department of Microbiology, Monash Medical Centre, 246 Clayton Road, Clayton, Victoria 3168. Australia; 5 Department of Microbiology, Austin Hospital, 145 Studley Road, Heidelberg, Victoria 3084, Australia; 6 Australian and New Zealand Intensive Care Research Centre, School of Public Health and Preventive Medicine, Monash University, 5 Commercial Rd, Prahran, Melbourne, Victoria 3181, Australia

## Abstract

**Introduction:**

To estimate the incidence of intensive care unit (ICU)-acquired bloodstream infection (BSI) and its independent effect on hospital mortality.

**Methods:**

We retrospectively studied acquisition of BSI during admissions of >72 hours to adult ICUs from two university-affiliated hospitals. We obtained demographics, illness severity and co-morbidity data from ICU databases and microbiological diagnoses from departmental electronic records. We assessed survival at hospital discharge or at 90 days if still hospitalized.

**Results:**

We identified 6339 ICU admissions, 330 of which were complicated by BSI (5.2%). Median time to first positive culture was 7 days (IQR 5-12). Overall mortality was 23.5%, 41.2% in patients with BSI and 22.5% in those without. Patients who developed BSI had higher illness severity at ICU admission (median APACHE III score: 79 vs. 68, *P *< 0.001). After controlling for illness severity and baseline demographics by Cox proportional-hazard model, BSI remained independently associated with risk of death (hazard ratio from diagnosis 2.89; 95% confidence interval 2.41-3.46; *P *< 0.001). However, only 5% of the deaths in this model could be attributed to acquired-BSI, equivalent to an absolute decrease in survival of 1% of the total population. When analyzed by microbiological classification, *Candida*, *Staphylococcus aureus *and gram-negative bacilli infections were independently associated with increased risk of death. In a sub-group analysis intravascular catheter associated BSI remained associated with significant risk of death (hazard ratio 2.64; 95% confidence interval 1.44-4.83; *P *= 0.002).

**Conclusions:**

ICU-acquired BSI is associated with greater in-hospital mortality, but complicates only 5% of ICU admissions and its absolute effect on population mortality is limited. These findings have implications for the design and interpretation of clinical trials.

## Introduction

Nosocomial bloodstream infection (BSI) is a serious and potentially preventable complication of hospitalization and has been estimated to be the eighth leading cause of death in the USA [1]. Critically ill patients are particularly vulnerable to hospital-acquired infections [2,3], which are two to seven times more common in the ICU [4-7] and can account for approximately half of all hospital-acquired BSI [8].

ICU-acquired BSI has been estimated to complicate between 1.2% and 6.7% of all ICU admissions [9-13], 4.4% to 6.8% of admissions of longer than 48 to 72 hours in duration [14-16] and have an incidence of between 5 and 19 per 1,000 patient days [9,11,15]. These infections have been associated with increased morbidity, mortality, and health care expenses [9,12-19]. As a consequence, considerable clinical and research activity has been focused on attempts to improve patient outcome by their prevention.

BSI is more common in patients who have surgery, are immunocompromised, develop multiorgan dysfunction, require mechanical ventilation or renal replacement therapy, and have greater illness severity on ICU admission [3,20,21]. Some critically ill patients may be genetically predisposed to both developing BSI and dying in hospital [22]. Thus BSI may be a marker of illness severity and pre-morbid condition as well as a direct contributor to adverse outcome. As a consequence, our ability to demonstrate the survival benefit of any intervention to prevent BSI will be dependent on the baseline incidence of BSI, the mortality rate of patients who develop it, and, crucially, on its true independent influence on outcome once correction has been made for other markers of illness severity.

Accordingly, we performed an observational study in a large cohort of critically ill patients and sought to estimate the incidence of BSI, the mortality rate of patients who acquire BSI, and its independent influence of mortality.

## Materials and methods

### Study population and data sources

We performed a retrospective observational analysis of the incidence of BSI acquired during ICU admission at two university-affiliated hospitals in Melbourne, Australia. Data were obtained from prospectively collected electronic databases of ICU admissions and hospital microbiology records of positive blood cultures. Standard protocols for the collection, analysis, and reporting of blood cultures were employed. Complete data were available for 11 years (Jan 1998 to Feb 2009) in one centre (Austin Hospital) and six years (Jan 2003 to Dec 2008) in the other (Monash Medical Centre). Local ethics committee approval was obtained for re-analysis of routinely collected data, waiving requirement for specific patient consent. Data were available on tip cultures from intravascular devices in the Austin Hospital allowing a sub-group analysis of proven catheter-associated BSIs in this cohort of patients (72% of the total population).

### Definitions

We used Center for Disease Control (CDC) definitions of ICU-acquired BSI (see Additional file 1) [23,24]; we considered both primary and secondary BSIs in our analysis. Nosocomial BSI in the ICU was defined as blood cultures taken in the presence of clinical evidence of infection for a bacterium or fungus obtained more than 72 hours after admission to the ICU. Thus, we included only those blood cultures taken after the third calendar day of ICU stay as reported by previous investigators [14,15,17,25].

Routine drawing of blood cultures was not ICU practice in the participating hospitals; we thus regarded all positive blood cultures obtained in the ICU as indicative of suspected infection.

In accord with CDC guidelines [24], we did not include cultures of coagulase-negative staphylococci or other common commensal skin organisms unless two cultures separately isolated the same species of microorganism. The first positive culture after the third ICU day was used to define the occurrence of ICU-acquired BSI. To allow study of a population at risk, we excluded all ICU admissions of less than 72 hours' duration.

BSI was considered to be catheter-associated if there was a positive tip culture from an intravascular device removed in the two days before or after the positive blood culture and the microbiological isolates from tip and blood were likely to represent the same infection (same species or compatible mixed growth).

### Aim

We sought to document the incidence of acquired BSI in our ICU populations and to obtain an estimate of its effect on subsequent survival. We hypothesized that although BSI is likely to be associated with greater risk of death in an individual its relative frequency in the total ICU population might limit its impact on overall mortality.

### Data analysis

We merged ICU admission and microbiology result databases and positive blood cultures paired with relevant ICU admission data by date and unique patient identifiers. Data were available on patient demographics, admitting specialty, duration of ICU admission, Acute Physiology and Chronic Health Evaluation III (APACHE III) physiology score on admission, APACHE III chronic health categories, need for mechanical ventilation or renal replacement therapy during ICU stay, and death during hospital admission.

To facilitate statistical analysis, APACHE III chronic health categories were used to define patient groups that might be at increased risk of BSI, namely: disseminated malignancy (metastatic cancer, lymphoma, leukemia, or myeloma), immunodeficiency (immunosuppression by illness or disease including HIV/AIDS), liver disease (hepatic failure and cirrhosis), chronic kidney disease, chronic pulmonary disease, and type 1 diabetes mellitus. Admission type was defined as surgical or non-surgical based on hospital admitting unit. Survival was defined as survival to hospital discharge or 90 days after ICU admission if the patient was still in hospital.

We performed univariate comparisons using *GraphPad Prism *version 5.0a for Mac OS (GraphPad Software, San Diego, California, USA [26]) and multivariate analysis and survival plots using *R: A language and environment for statistical computing *(R Foundation for Statistical Computing, Vienna, Austria [27]) utilizing the packages *survival *[28] and *Design *[29]. Categorical data were reported as percentages, and compared using the chi squared test with Yates' correction. Continuous data were reported as median with inter-quartile range (IQR) and compared using the Mann-Whitney U test with Gaussian approximation. For comparisons, statistical significance was denoted by two-sided *P *values of less than 0.05.

Baseline risks for ICU-acquired BSI were examined in a multivariate logistic regression analysis with backward elimination of non-significant predictor variables. Significance was assessed against the null model by chi squared test of residual deviance, goodness-of-fit by unweighted sum of squares test, and predictive ability by calculation of the c-statistic. Independent predictors of survival were modeled using a Cox proportional-hazard analysis. As ICU-acquired BSI was not present at baseline it was incorporated into the model as a time-dependent co-variate [30], other factors were either present at ICU admission (admission illness severity, demographics, and comorbidities) or, in the vast majority of cases, were initiated in the first 72 hours of ICU stay (mechanical ventilation and renal replacement therapy) and were treated as time-independent co-variates. We avoided the need to consider hospital discharge as a competing endpoint by deeming all patients discharged alive from hospital to have survived to day 90 for the purposes of the survival analysis rather than censoring them at time of discharge. The proportional-hazard assumption was assessed by inspection of Schoenfeld residual plots. A Cox proportional-hazard analysis was repeated to separately assess the independent effect of the five most common microbiological diagnoses, grouping similar organisms to preserve statistical power. Similarly, in the cohort of patients from the Austin hospital, we modeled the relative effect on survival of catheter-associated and non-catheter-associated BSI.

## Results

**Figure 1:**
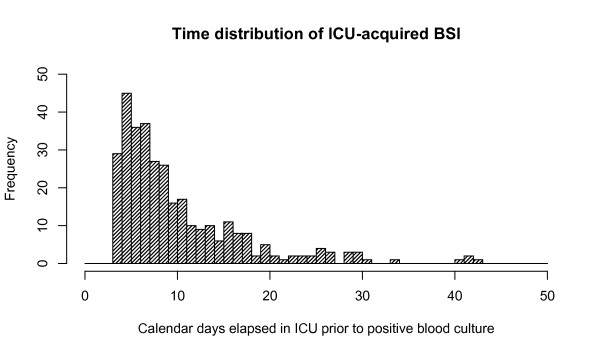
**Histogram of time of diagnosis of ICU-acquired BSI**. Due to uncertainty over the exact time at which blood cultures were taken, some taken in the fourth calendar day in the ICU (first column) might have in fact been taken between 48 and 72 hours after ICU admission. At the most 29 patients may have been miss-attributed. Conversely, use of a later cut-off might exclude a similar number of genuine ICU-acquired BSI. Analysis was designed to err on the side of maximal inclusion. BSI, bloodstream Infection.

We studied 6,339 ICU admissions of more than 72 hours' duration. ICU-acquired BSI complicated 5.2% of these admissions (Table 1) and 9.5 new BSIs were acquired in the ICU per 1,000 patient days at risk. Median time to first positive blood culture in those acquiring BSI was seven days (IQR 5-12; Figure 1). Microbiological classification of ICU-acquired BSI is shown in Table 2.

**Table 1:** Characteristics of patients admitted to ICU for 72 hours or longer with univariate comparisons

	All Admissions	No Acquired BSI	Acquired BSI	*P*
Number of Admissions	6,339	6,009 (94.8%)	330 (5.2%)	
APACHE III (admission)	68 (52-88)	68 (52-87)	78.5 (61-97)	< 0.001
Age	64.9 (50-75)	65.0 (46-75)	62.6 (49-73)	0.02
Male Sex	62.1%	61.9%	65.2%	0.26
Surgical Admission	55.5%	55.4%	57.6%	0.48
Mechanical Ventilation	69.2%	69.2%	70.3%	0.79
Renal Replacement Therapy	6.7%	6.2%	15.2%	< 0.001
Immune Deficiency	7.2%	7.0%	10.6%	0.02
Malignancy	15.1%	14.8%	19.1%	0.04
Liver Disease	7.0%	6.7%	12.4%	< 0.001
Chronic Kidney Disease	3.8%	3.7%	4.8%	0.36
Chronic Pulmonary Disease	6.5%	6.6%	5.8%	0.64
Insulin-Requiring Diabetes	2.8%	2.7%	3.9%	0.27
ICU LOS (days)	6 (4-10)	5 (4-9)	15 (10-25)	< 0.001
Hospital Mortality	23.5%	22.5%	41.2%	< 0.001

For continuous variables median and inter-quartile range are shown.

APACHE, Acute Physiology and Chronic Health Evaluation; BSI, bloodstream infection; LOS, length of stay.

**Table 2:** Microbiological isolates during 330 ICU admissions complicated by acquired bloodstream infection

Microbiological Isolate	Percentage of Admissions with BSI
	
Gram negative Bacilli	28.2%
*Staphylococcus aureus*	26.7%
Coagulase-negative staphylococci	24.3%
Enterococci	17.0%
*Candida *species	15.5%
Others	6.7%

In 44 admissions more than one species was isolated

BSI, bloodstream infection.

### Univariate analysis

Univariate analysis is presented in Table 1. BSI was associated with an 18.7% increase in crude hospital mortality from 22.5% to 41.2%. However, as BSI was infrequent, crude mortality in the total population was 23.5%, only 1% greater than in patients who did not acquire BSI (22.5%). This difference in mortality represents an unadjusted population attributable risk percentage [31] of 4.3% - that is, before adjusting for confounding variables, 4.3% of all deaths could be attributed to excess mortality after acquired-BSI.

**Figure 2:**
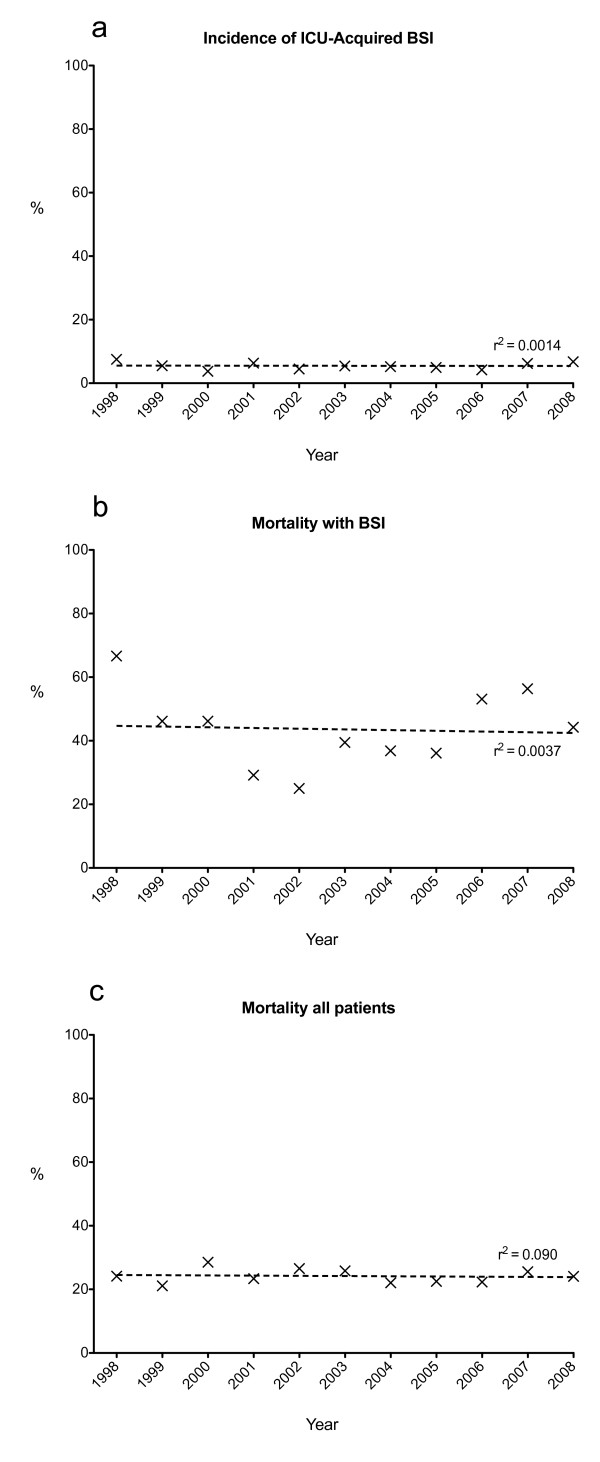
**Year on year trend in incidence of BSI (Panel a), mortality in patients with BSI (Panel b) and all ICU admissions of longer than 72 hours (Panel c) showing no significant trend in change in these variables over the study period**. BSI, bloodstream Infection.

During each full year of the study, rates of BSI varied from 4.4% to 8.1%, overall mortality from 21.1% to 26.5% and crude mortality in patients with ICU acquired BSI from 25% to 66%. However, there were no trends toward a systematic alteration in these frequencies over time (Figure 2).

Patients who developed acquired-BSI had greater ICU length of stay than those who did not (median 15 days vs. 5 days; *P *< 0.001). Patients acquiring BSI were also significantly sicker at ICU admission and had more co-morbidities (Table 1). Overall, 49 patients who developed BSI in the ICU were alive and in hospital 90 days after ICU admission and were treated as survivors in our analysis. Of these, only three subsequently died late during their hospital stay, a rate similar to the overall population.

### Prediction of BSI

We examined risks for ICU-acquired BSI by developing a logistic-regression model for its prediction (Table 3). In this model, only higher APACHE III scores, the need for renal replacement therapy, liver disease, and surgical admission were risk factors for acquisition of BSI, whereas older age lessened the odds of a diagnosis of BSI. The model was significantly better than a null model (*P *<< 0.001); however, its predictive ability was poor with a *c-statistic *of 0.63, implying that factors beyond the baseline predictors examined had a large influence on the development of BSI in the ICU.

**Table 3:** Logistic regression analysis of risk factors for acquired bloodstream infection

Risk Factor	Odds Ratio	95% CI	*P*
APACHE III (factor/point)	1.01	1.01-1.02	< 0.001
Age (factor/year*)	0.99	0.98-1.00	< 0.001
Renal Replacement Therapy	2.16	1.54-2.99	0.002
Liver Disease	1.45	1.00-2.06	0.041
Surgical Admission	1.28	1.02-1.63	0.037

APACHE, Acute Physiology and Chronic Health Evaluation; BSI, bloodstream infection; CI, confidence interval.

*Odds ratio of 0.99/year equates to a 0.9 times change in odds of BSI with a 10 year increase in age.

### Survival analysis

**Figure 3:**
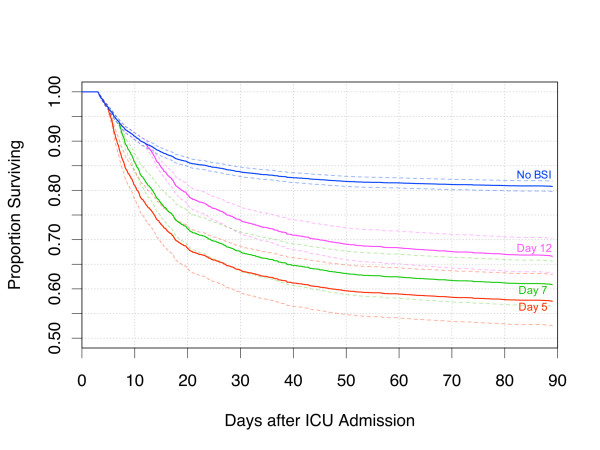
**The independent effect of acquired BSI on hospital mortality in a Cox-proportional model of survival after ICU admission of 72 hours or longer**. Plots show predicted survival in the absence of acquired bloodstream Infection (BSI) and with BSI occurring at the median time (day 7) and the lower and upper quartiles for time of acquisition (days 5 and 12). All other covariates fixed at population means. Dotted lines show 95% confidence limits.

**Figure 4:**
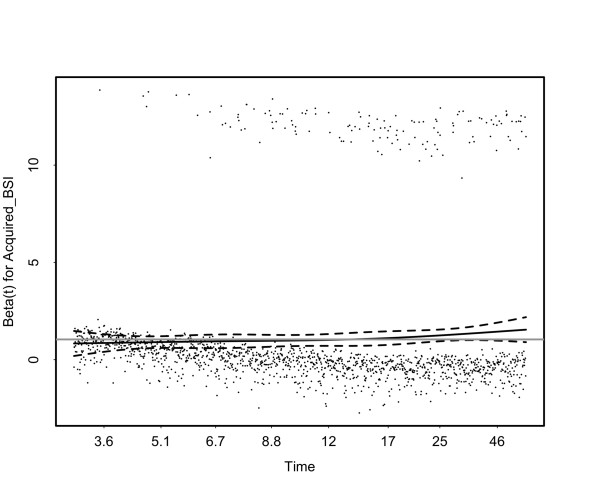
**Plot of scaled Schoenfeld residuals versus transformed time (based on Kaplan-Meir estimate of survival function) demonstrating acceptable linearity for the proportional hazard for the covariate Acquired BSI**. Beta(t) is the exponential associated with the covariate, equivalent to the natural logarithm of the hazard ratio. The solid black line is a smoothing-spline fit to the plot, with the broken lines representing a ± 2-standard-error band around the fit. Grey line represents a completely proportional (time-invariant) hazard ratio of 2.89. BSI, bloodstream Infection.

Controlling for baseline difference in a Cox proportional hazard model, we confirmed that BSI was associated with increased risk of death, with a hazard ratio for death from the time of acquisition of BSI of 2.89 (Table 4). Acquired BSI infection was modeled as a time-dependent covariate and the effect on actual survival in the model was thus dependent on the time of acquisition of BSI (Figure 3). This model is dependent on the validity of the proportional hazard assumption for acquired BSI and inspection of residual plots confirmed this was reasonable over the timescale in question (Figure 4). Accordingly for an individual, BSI occurring at day seven (median time of acquisition) was associated with an approximate 20% absolute increase in hospital mortality compared with absence of BSI, when all other baseline hazards were held at population means.

**Table 4:** Cox-proportional hazard analysis for hospital survival

Risk Factor	Hazard Ratio	95% CI	*P*
APACHE III (factor/point)	1.02	1.02-1.02	< 0.001
Age (factor/year*)	1.01	1.01-1.01	< 0.001
Acquired BSI	2.89	2.41-3.46	< 0.001
Surgical Admission	0.78	0.71-0.87	< 0.001
Liver Disease	1.34	1.12-1.61	0.001
Malignancy	1.23	1.08-1.40	0.002
Mechanical Ventilation	1.13	1.00-1.26	0.041
Immune Deficiency	1.18	0.99-1.40	0.059
Chronic Kidney Disease	1.18	0.94-1.48	0.148
Chronic Pulmonary Disease	1.15	0.95-1.39	0.162
Insulin-Requiring Diabetes Mellitus	1.18	0.90-1.54	0.221
Renal Replacement Therapy	1.09	0.92-1.30	0.334
Male Sex	0.98	0.89-1.09	0.767

APACHE, Acute Physiology and Chronic Health Evaluation; BSI, bloodstream infection; CI, confidence interval.

*Hazard ratio of 1.01/year equates to a 1.1 times hazard for death with a ten year increase in age.

**Figure 5:**
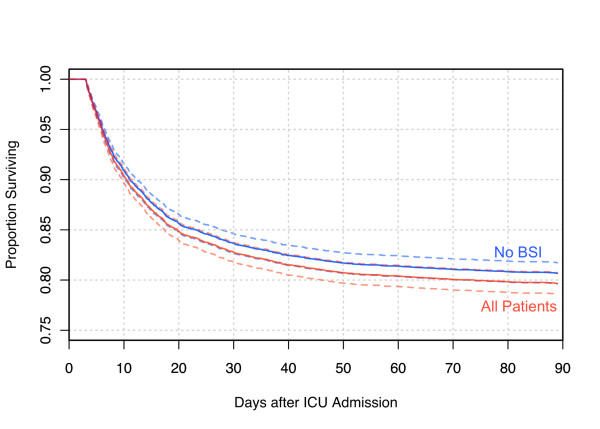
**Survival in the Cox-proportional hazard model in the absence of acquired BSI and in the whole population of ICU admissions lasting 72 hours or longer**. In this model, at the observed incidence of acquired bloodstream Infection (BSI) in the whole population, only a 1% increase in total hospital mortality can be associated with BSI. All other covariates fixed at population means. Dotted lines show 95% confidence limits.

In Figure 5, we modeled the survival effect of all BSI, occurring at the rate and times of acquisition observed in the whole population, comparing against a group not acquiring BSI, with all other baseline hazards held at population means. In this model, the population attributable risk of death at or before day 90 was 4.95% and excess mortality in the entire study population, associated with the observed occurrence of acquired-BSI and independent of the other baseline hazards, was 1%.

### Effect of microbiological diagnosis

We separately repeated our survival analysis to model the effect of the five most common classes of BSI on outcome (Table 5). In this analysis enterococci and coagulase-negative staphylococci infections were not significantly associated with survival while *Staphylococcus aureus *and Gram negative infections both approximately doubled the risk of death and candidemia was associated with an over four-fold risk of dying in hospital.

**Table 5:** Cox-proportional hazard analysis for effect of microbiological diagnosis on hospital survival (only microbiological co-variates are shown)

Microbiological Isolate	Percentage of all admissions	Unadjusted mortality	Hazard Ratio	95% CI	*P*
*Candida *species	0.8%	69%	4.60	3.23-6.57	< 0.001
Gram negative Bacilli	1.5%	38%	2.13	1.49-3.04	< 0.001
*Staphylococcus aureus*	1.5%	42%	2.07	1.48-2.90	< 0.001
Enterococci	0.9%	34%	1.49	0.93-2.39	0.10
Coagulase-negative staphylococci	1.3%	28%	1.23	0.78-1.94	0.36
None	94.8%	23%	-	-	-

CI, confidence interval.

### Catheter-associated BSI

We assessed the possibility of catheter associated bloodstream infection (CABSI) in 167 of 330 cases of BSI, in 127 of these an intravascular device tip was sent for culture in the two days either before or after a positive blood culture. In 34 cases (20.4% of BSI), a positive tip culture with a compatible microorganism identified likely CABSI. CABSI microbiological isolates and types of intravascular devices involved are shown in Tables 6 and 7, respectively. Univariate comparison between catheter-associated and non-catheter-associated BSI demonstrated a non-significant trend toward lower hospital mortality with CABSI (32.4% vs. 44.4%; *P *= 0.25; Table 8); however, in a Cox proportional-hazard analysis of all Austin Hospital patients, the hazard ratio for in-hospital death before 90 days linked with CABSI was 2.64 (95% confidence interval (CI) = 1.44-4.83; *P *= 0.002 vs. no BSI; Table 8) - not significantly different from the hazard associated with non-CABSI (hazard ratio = 3.18; 95% CI = 2.43-4.17; *P *< 0.001 vs. no BSI; *P *= 0.57 vs. catheter-associated BSI; Table 8).

**Table 6:** Microbiological diagnosis in catheter-associated bloodstream infection (BSI) and non-catheter-associated BSI from 167 patients at Austin Hospital

	Non-catheter-associated BSI	Catheter-associated BSI
*Staphylococcus aureus*	21.8%	17.7%
Coagulase negative staphylococci	16.5%	23.5%
Enterococci	13.5%	11.8%
Gram negative Bacilli	24.8%	44.1%
*Candida *species	18.8%	5.8%

No significant difference in distribution of microbiological isolates between groups, chi-squared test: *P *= 0.14.

**Table 7:** Intravascular devices associated with proven ICU-acquired, catheter-associated bloodstream infection during ICU admissions at Austin Hospital

Intravascular Device	n (%)
Central venous catheter	16 (46%)
Dialysis catheter (non-tunnelled)	7 (20%)
Pulmonary artery catheter	6 (17%)
Peripherally inserted central catheter	2 (6%)
Arterial catheter	2 (6%)
Tunnelled central venous catheter	1 (3%)
Temporary pacing wire	1 (3%)

**Table 8:** Characteristics and hospital mortality in patients with microbiological evidence of catheter-associated bloodstream infection (BSI) versus positive blood cultures with no contemporaneous proven catheter infection (median and inter-quartile range for univariate comparison, hazard ratio (HR) with 95% confidence interval for Cox analysis)

	Catheter-associated BSI	Non catheter-associated BSI	*P*
Number (% of BSI)	34 (20.4%)	133 (79.6%)	
*Univariate comparison*			
APACHE III (admission)	66 (50-91)	72 (54-93)	0.41
Age (years)	59 (39-67)	61 (45-73)	0.32
ICU LOS (days)	17.5 (13-27)	15 (9-23)	0.21
Hospital Mortality	32.4%	44.4%	0.25
*Cox hazard analysis*			
HR for death in hospital	2.64 (1.44-4.83)	3.18 (2.43-4.17)	0.57

APACHE, Acute Physiology and Chronic Health Evaluation; LOS, length of stay.

Data from Austin Hospital patient cohort only. Cox proportional-hazard analysis incorporated all covariates used in Table 4.

## Discussion

### Statement of main findings

We studied 6,339 admissions of greater than 72 hours in two university-affiliated ICUs. We found that ICU-acquired BSI complicated approximately 1 in 20 of these admissions and that increased illness-severity, surgery, immunological compromise, liver disease, mechanical ventilation, and renal replacement therapy predicted its occurrence. We further found that BSI was independently associated with a close to three-fold increased risk of death from the time of positive blood culture and that the proportional hazard assumption was robust. This implies that although the proportional effect of BSI on mortality is constant over time, if BSI occurs early in ICU admission, when the baseline rate of death is high, the absolute effect on chance of survival is greater than if it occurs later in the course of critical illness. We note, however, that residual cofounders are likely to exist outside of our statistical analysis, a suspicion supported by the weak ability of the logistic-regression model to predict the development of ICU-acquired BSI based on the presence of the baseline predictors available. This suggests that our assessment of the impact of acquired-BSI on survival should be regarded as an upper-estimate of any effect.

We also found that the collective effect of BSI on survival was statistically related to infections with candida, *S. aureus*, and Gram-negative bacilli, while infections with coagulase-negative staphylococci and enterococci were not significantly associated with increased risk of death in our dataset. In the cohort of patients from the Austin Hospital, BSI identified as likely catheter-associated remained a significant hazard for non-survival, and risk of death was not significantly lower than that related to non-catheter-associated BSIs.

Together these findings imply, that, because of the low incidence and the estimated independent contribution to mortality, the totality of BSI accounted for, at most, an additional 1% excess mortality in the entire ICU population. This adjusted effect of BSI is very close to the unadjusted effect of BSI on survival. This may be because, although patients with acquired BSI were sicker and had more co-morbidities, they had to survive a certain length of time in order to be able to be diagnosed and categorized with BSI. In our model these competing effects appear to offset each other leading to the similarity of the adjusted and unadjusted survival.

### Relation to previous findings

Our incidence of BSI is similar to that reported in other observational studies [9,11-15]. This similarity suggests that our findings may have external validity. Our mortality findings are also in agreement with previous studies of mortality among patients with BSI [9,11-14,16,19,32,33] with an increased risk of death of about three- to four-fold [14,16]. Other investigators found a lesser impact of BSI on mortality [10,34-41] suggesting that the impact of BSI may vary with the setting and methodology.

Our finding the infection with coagulase-negative staphylococci did not confer significant additional risk of death is in accord with findings that catheter-related BSIs are less strongly associated with risk of death [16,42,43]; however, this was not borne out in our analysis of BSI with evidence of catheter infection. This suggests that the virulence of the microorganism rather than the source of infection may be more important in determining outcome.

Finding an association between illness severity and incidence of BSI in the ICU is in keeping with previous reports [9,11-14]. Similarly need for renal replacement therapy in the ICU is a documented risk factor for BSI [44]. The negative association between increasing age and decreased risk of developing BSI has also been observed previously [45]. Older age may be associated with a less pronounced inflammatory response to infection [46], consequently, the likelihood of blood culture sampling may be lower, reducing observed incidence of BSI in older patients.

### Significance of study findings

Our study expands current knowledge of the incidence and independent impact of ICU-acquired BSI on survival. First, it suggests that the likely typical incidence of acquired BSI in our ICU population is approximately 5%. This observation is a helpful comparator for control groups in interventional trials aimed at reducing BSI. This finding also suggests that conclusions from studies where the "control" incidence of acquired BSI is higher than this may not be directly transferable to all ICUs. Second, it confirms that some ICU-acquired BSI likely contributes independently to an increased risk of death and that prevention of these infections (predominantly *S. aureus*, Gram negative and *Candida*) is an important therapeutic goal. Third, it suggests that catheter-associated BSIs are clinically significant and their prevention is also of importance. Finally, it identifies several important factors that are associated with increased risk of developing BSI. However, our study also indicates that our ability to predict its development using baseline characteristics and major interventions (surgery, renal replacement therapy, and mechanical ventilation) remains limited. Thus, from the data routinely available early on in ICU admission, identification of a sub-group of higher risk patients for BSI-preventive intervention appears difficult. This observation is important, because it suggests that more research is required to identify patient characteristics, beyond those conventionally collected near ICU admission, to allow better prediction of risk of nosocomial infection. Better predictive models might allow appropriate targeting of cumbersome or costly preventative interventions and enhance the power of clinical studies examining such interventions.

The finding that BSI was independently associated with a three-fold increased risk of death from the time of positive blood culture implies that although the proportional effect of BSI on mortality is constant over time, if BSI occurs early in ICU admission, when the baseline rate of death is high, the absolute effect on chance of survival is greater than if it occurs later in the course of critical illness. We note, however, that residual cofounders are likely to exist outside of our statistical analysis, a suspicion supported by the weak ability of the logistic-regression model to predict the development of ICU-acquired BSI based on the presence of the baseline predictors available. This suggests that our assessment of the impact of acquired-BSI on survival should be regarded as an upper-estimate of any effect.

Finally, because of the relatively low incidence of ICU-acquired BSI in our population, ICU-acquired BSI would account for only 5% of deaths, that is 1% excess mortality in the total population. This observation has several implications for randomized controlled trials of interventions aimed at decreasing mortality by preventing ICU-acquired BSI. For example, a putative untargeted intervention capable of preventing 50% of ICU-acquired BSI would be expected to reduce the overall mortality of ICU patients staying for longer than 72 hours by only 0.5%. Accordingly, even a very effective intervention would have to be administered to 200 patients to save one life. This effect is impossible to test in any feasibly sized randomized controlled trial (in excess of 100,000 patients would be required for adequate power). Interventions to prevent BSI could have a greater impact on survival by impacting sub-clinical, undiagnosed, or localized infection. However, the strength of such effects would need to be quantified if investigators wish to be assured that interventional studies were adequately sized. Our data do suggest that use of formally diagnosed BSI as a surrogate or secondary endpoint in untargeted interventional studies may not be feasible.

### Study strengths and weaknesses

This study has several strengths. We used a large sample of patients. Data were collected by dedicated data collectors and electronically stored and were thus not amenable to manipulation or bias. Similarly, microbiological data were collected as part of patient care. We assessed the independent contribution of BSI to patient outcome, providing useful information for trial design and for the assessment of the relevant interventional literature.

On the other hand, our study also has some weaknesses. Its findings may not be directly generalizable to differing microbiological environments worldwide. However, its results are comparable with those in similar studies conducted in the USA and Europe suggesting a degree of external validity. During the 11-year study period changes in case-mix, clinical workload, and clinical practice could have affected incidence and outcome of ICU-acquired BSI. However, no trend was evident on inspection of the yearly data (see Additional file 1). By examining this time-span we were able to include data from over 6,000 ICU admissions making this one of the largest studies of BSI in intensive care.

We did not have detailed clinical information including exact trigger for drawing blood cultures, antimicrobial therapy, response to treatment, and cause of death. Nor could we determine whether individual episodes of BSI represented true infections. However, we excluded commensal skin organisms isolated in single blood culture bottles, making it more likely that our isolates represented true BSI. The association of such BSI with illness severity, invasive interventions, and mortality all support this notion. Furthermore, although exclusion of a small number of non-clinically significant infections might increase the attributable-mortality of BSI, this would also decrease the observed incidence of BSI and would thus be unlikely to substantially alter our estimate of the effect of BSI on overall survival.

Our analysis of catheter-associated BSI was confined to only one study centre. We required a positive tip culture to confirm a likely catheter source. Thus, we may have missed some catheter-related infection although frequency of catheter-associated infection in our cohort (about 20%) was similar to that reported by some previous investigators [9,14]. Conversely, in a few patients, the catheter might have been secondarily infected by blood-borne infection. However, by identifying a group of patients with likely catheter-associated infection, we were able to demonstrate that increased risk of death remained significant in patients where a catheter source of infection was very likely.

We did not compare patients developing BSI with a matched control population. However, such retrospective matching of controls is always approximate and susceptible to unmeasured effects. In our study, without day-to-day clinical data on patients, we were limited to adjusting for baseline factors and major interventions (such as mechanical ventilation) usually commenced early in ICU admission. We also did not assess the effect of BSI on duration of ICU stay. However, the direction of causality is very difficult to determine, because greater length of exposure to risk will tend to increase the incidence of BSI, while, at the same time, occurrence of BSI will tend to delay ICU discharge. Given the inability to assess direction of causality, we did not attempt to incorporate ICU length of stay into our statistical models. We note that infections with coagulase-negative staphylococci, which would be expected to be more common with greater length and complexity of ICU stay, were not significantly associated with risk of death. This suggests that the associations seen with mortality for other microorganisms are likely to be causative. However, because of concerns about unmeasured confounders, our estimate of attributable-mortality from ICU-acquired BSI should be regarded as an upper estimate of any effect. Significantly, however, even using this high estimate of attributable-mortality, BSI had little impact on the overall survival of the total population, contributing to, at most, an absolute 1% increase in hospital mortality.

## Conclusions

In a study of over 6,000 ICU admissions lasting longer than 72 hours, ICU-acquired BSI was associated with a doubling in risk of death in hospital to approximately 40%. This correlation remained even after adjustment for baseline illness severity, demographics, and co-morbidities in a Cox proportional hazard model and was almost entirely attributable to BSI with significant pathogens. However, ICU-acquired BSI was uncommon thus, although of great clinical impact to those individuals affected by it, its attributable excess mortality could be, at most, 1% of the total population. This effect implies that a) the survival benefit of untargeted interventions aimed at reducing the rate of proven ICU-acquired BSI would be undetectable in any practically sized controlled trial; b) claims of improved survival from interventions aimed at reducing acquisition of BSI in the ICU should be treated with caution.

## Key messages

• Acquired BSI is independently associated with significantly increased risk of death in critically ill patients.

• This association persists for catheter-associated BSI.

• These infections are relatively uncommon so that, despite significance to individuals, their contribution to overall mortality in an unselected population of ICU patients is small.

## Abbreviations

APACHE: Acute Physiology and Chronic Health Evaluation; BSI: bloodstream Infection; CABSI: catheter-associated bloodstream infection; CI: confidence interval; IQR: inter-quartile range.

## Competing interests

The authors declare that they have no competing interests.

## Authors' contributions

JRP, JEE, EVL and RB conceived the study and devised the data analysis plan. JRP and JEE performed background literature review. GCT, TMC, TMK, GKH, PDRJ and BCM collected the primary datasets. NS collected additional data on catheter-associated infection. JRP, JEE, EVL, NS and GCT performed data analysis. JRP performed statistical analysis and wrote the manuscript. All authors then reviewed the draft and had input to revision of the final manuscript.

## Supplementary Material

Additional file 1 **Box 1**. CDC/NHSN surveillance definition of health care-associated infection. LCBI, Laboratory-confirmed primary bloodstream infection [24]. Click here for file
