# Supplementary material for: Acquired bloodstream infection in the intensive care unit: incidence and attributable mortality
Source: Crit Care. 2011 Mar 21;15(2):R100. doi: 10.1186/cc10114 (PMC3219371; doi:10.1186/cc10114)
Supplement: Additional file 1 — Box 1. CDC/NHSN surveillance definition of health care-associated infection. LCBI, Laboratory-confirmed primary bloodstream infection [24]. [file cc10114-S1.DOC]

**Box 1.** CDC/NHSN surveillance definition of health care–associated infection LCBI-Laboratory-confirmed primary bloodstream infection .

1.Patient has a recognized pathogen cultured from 1 or more blood cultures and organism cultured from blood is not related to an infection at another site*.

2. Patient has at least 1 of the following signs or symptoms: fever, chills, or hypotension and signs and symptoms and positive laboratory results are not related to an infection at another site and common skin contaminant (ie, diphtheroids [Corynebacterium spp], Bacillus [not B anthracis] spp, Propionibacterium spp, coagulase-negative staphylococci [including S epidermidis], viridans group streptococci, Aerococcus spp, Micrococcus spp) is cultured from 2 or more blood cultures drawn on separate occasions.

**We did not consider whether infection was related to infection at another site as we wished to include primary and secondary (ie catheter-associated) BSI.*
